# The complete chloroplast genome of *Angelonia angustifolia* Benth. (Plantaginaceae), an ornamental and medicinal plant

**DOI:** 10.1080/23802359.2022.2152642

**Published:** 2022-12-07

**Authors:** Jianhua Yue, Yan Dong, Yun Teng, Shoufu Gong

**Affiliations:** a School of Horticulture, Xinyang Agriculture and Forestry University, Xinyang, P. R. China; b School of Forestry, Xinyang Agriculture and Forestry University, Xinyang, Henan, P. R. China

**Keywords:** *Angelonia angustifolia* Benth., chloroplast genome, phylogeny, Plantaginaceae

## Abstract

In this study, we assembled and characterized the complete chloroplast (cp) genome of *Angelonia angustifolia* Benth., 1846, a herbaceous and perennial plant, native to Latin America. It is an ornamental and medicinal plant that showed bright prospects for application. The cp genome of *A. angustifolia* has a typical conserved quadripartite structure of 154,316 bp in total length. The genome includes a large single-copy (LSC) region (84,110 bp), a small single-copy (SSC) region (15,950 bp), and a pair of inverted repeat (IR) regions (27,128 bp). The cp genome contains 130 genes comprising 85 protein-coding, 37 tRNA, and 8 rRNA genes. Phylogenetic analysis indicates that *A. angustifolia* is closely related to *Bacopa monnieri*, *Scoparia dulcis*, and *Limnophila sessiliflora* in the Plantaginaceae. Taken together, the complete cp genomes of *A. angustifolia* provided significant insights and important information for molecular biology, evolution, and taxonomy in the genus *Angelonia*.

There are now more than 26 species in the genus *Angelonia*, many of which are utilized to study plant modeling, flower form, colors, and floral scent volatiles (Martins et al. 2014; Katsu et al. 2017; Ghissing and Mitra 2021). One of these species, *Angelonia angustifolia* Benth. (first mentioned in 1846, https://florida.plantatlas.usf.edu/) is a herbaceous, perennial plant native to Latin America (Deyrup et al. 2014). This species is resistant to heat temperature but is cold-sensitive (Seaton et al. 2014). This species is well-known for its high ornamental and medicinal value (Deyrup et al. 2014). *Angelonia angustifolia* is commonly used as potted plant for landscaping (Blanchard and Runkle 2011; Deyrup et al. 2014). It contains numerous medicinal active ingredients such as anti-inflammatory metabolites (Deyrup et al. 2014; Katsu et al. 2017). However, the systematic position of the genus *Angelonia* was uncertain (APG 2003, 2009). Over the past several decades, the genus *Angelonia* has been placed in the Scrophulariaceae family and Plantaginaceae family (Vogel and Machado 1991; Martins et al. 2014). The chloroplast (cp) genome has been used to investigate the developmental and phylogenetic information of plants because of its maternal inheritance and conserved structure (Wang et al. 2018). In this present study, the complete cp genome of *A. angustifolia* was assembled and analyzed to better understand the phylogenetic position of *A. angustifolia*.

*Angelonia angustifolia* leaf specimens were sampled from Xinyang, Henan Province, China (the experimental base of Xinyang Agriculture and Forestry University: 114°12′ E, 32°16′ N, altitude: 102 m). Afterwards, these specimens (Bio-sample accession: SAMN25210007) were stored at −80°C of the Horticultural Plant Biotechnology Laboratory. A specimen was deposited at the Herbarium of the Horticultural Plant Biotechnology Laboratory, Xinyang Agriculture and Forestry University (contact Jianhua Yue, jhyues@163.com) under the voucher code XAA2201102. Genomic DNA was extracted by the CTAB method (Odahara et al. 2009). After the DNA extraction from leaf tissues, the DNA sample was sent to Shanghai Origingene Biotechnology Co., Ltd. (Shanghai, China) to construct a DNA library. Then, the DNA library was sequenced by using the Illumina NovaSeq 6000 sequencing platform (Illumina, San Diego, CA, USA). Approximately, 7.8 GB of raw data was generated with 150 bp paired-end read lengths. Raw reads in fastq format were filtered using Trimmomatic v0.39 with default parameters retrieving the clean data (Bolger et al. 2014). The data were *de novo* assembled using NOVOPlasty based on clean reads (Dierckxsens et al. 2016). Then, to check the accuracy of assembly results, the slimmed assembly graph and selected target assembly graph were visualized by Bandage (Wick et al. 2015). The cp genome annotation was performed by Geneious v 11.1.5 (Biomatters, Auckland, New Zealand) (Kearse et al. 2012).

The complete cp genome of *A. angustifolia* has a typical conserved quadripartite structure of 154,316 bp in length with an overall GC content of 37.66%, containing four distinct regions: an large single-copy (LSC) region (84,110 bp), an small single-copy (SSC) region (15,950 bp), and a pair of inverted repeat (IR) regions (27,128 bp). The complete cp genome consists of 130 genes, comprising 85 protein coding, 37 tRNA, and 8 rRNA genes. The average coverage of the cp genome reached ×27,297 sequencing depth.

To explore the phylogenetic relationship of *A. angustifolia* with its closely related species, a phylogenetic tree was constructed based on the cp genome of 16 species which were downloaded from NCBI GenBank. The sequences were aligned by MAFFT v7.307 using regular settings (Katoh and Standley 2013), and MEGA X was used to construct the phylogenetic tree (Kumar et al. 2018). The robustness of the topology was calculated using the maximum-likelihood method. The program operating parameters were set as follows: a Tamura-Nei nucleotide substitution model with 1000 bootstrap replicates, accompanied by Gamma distributed with Invariant site (G + I) rates, and partial deletion of gaps/missing data according to Nguyen et al. (2015). Phylogenetic analysis results strongly supported that *A. angustifolia* was fully resolved in a clade with *Bacopa monnieri*, *Scoparia dulcis*, and *Limnophila sessiliflora* in the Plantaginaceae family (Figure 1). The analysis of the cp genome of *A. angustifolia* may provide a theoretical basis for genetic breeding, as well as determining phylogenetic relationships of *A. angustifolia* with related species.

**Figure 1.**
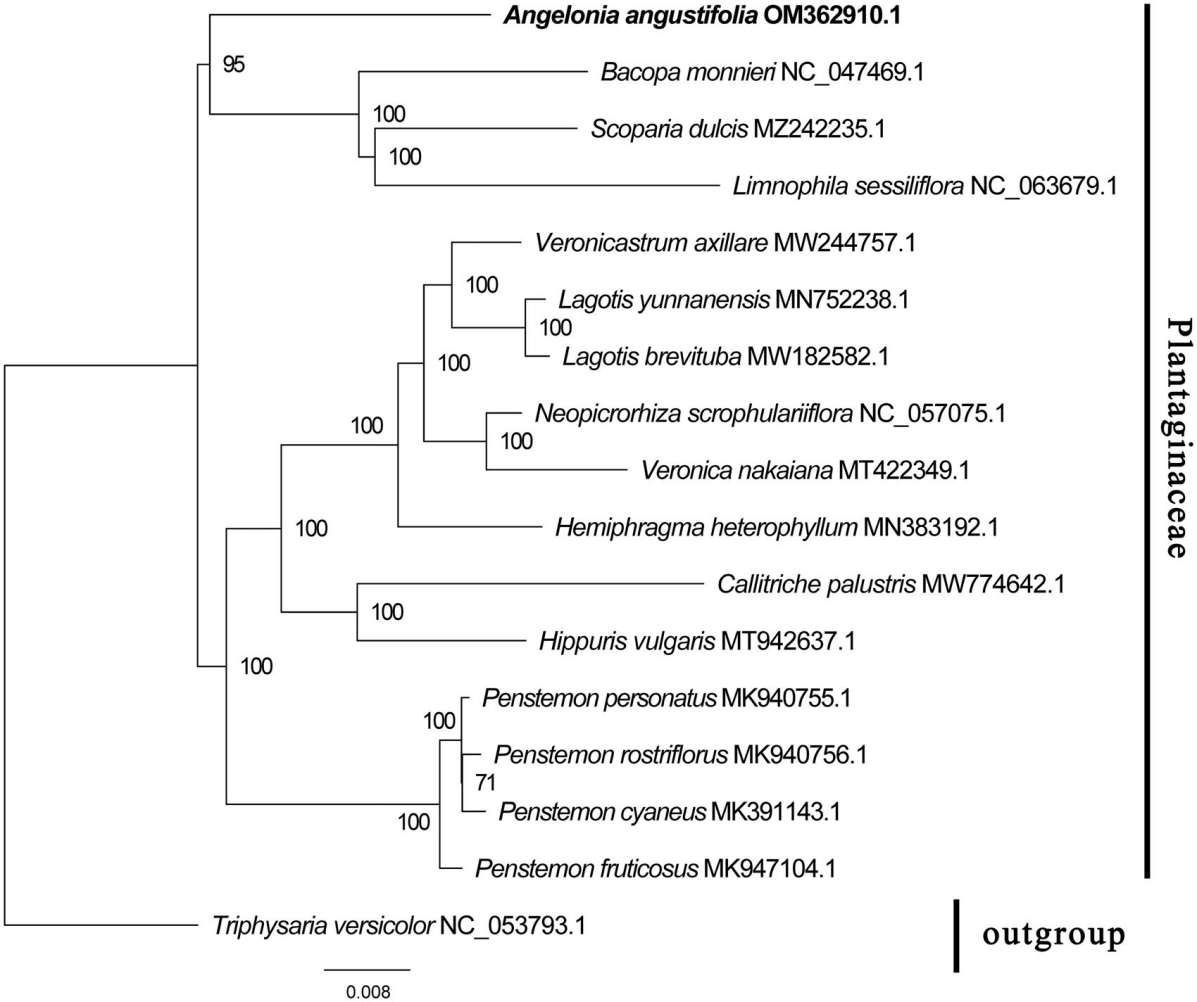
Phylogenetic analysis based on the complete cp genomes. The bootstrap values were shown on the nodes, the species, and GenBank accession number were shown at the end of each branch.

## Data Availability

The data that support the findings of this study are openly available in GenBank of NCBI at https://www.ncbi.nlm.nih.gov/. The complete cp genome has been deposited in GenBank under the accession no. OM362910. The associated Bio-project, SRA, and Bio-sample numbers are PRJNA799816, SRX13877377, and SAMN25210007, respectively.
